# Evaluation of epidemiological pattern and survey of incidence rate of Accidents and Incidents in the Khomein City, Markazi province

**Published:** 2019-08

**Authors:** Yasan Kazemzadeh, Ali Toolabi, Farshad Nadri, Azam Ahmadi, Rohullah Amini, Maryamsadat Shojaii

**Affiliations:** ^*a*^Khomein Faculty of Medical Sciences, Khomein, Iran.; ^*b*^Bam University of Medical Sciences, Bam, Iran.; ^*c*^Kermanshah University of Medical Sciences, Kermanshah, Iran.

## Abstract

**Background:**

Accidents and injuries are one of the most important causes of disability and death in developed and developing countries. Awareness of the dimensions and causes of accidents and injuries can be beneficial for health policymakers. Therefore, this study was conducted to aim of estimation the incidence rate and evaluation of epidemiological pattern of accidents and incidents in the Khomein City.

**Methods:**

This descriptive-analytical study from cross-sectional type was carried out using data from the national program of accidents and injuries registry in the Khomein Province in 2017. The study population is injured by accidents referring to the hospital. To calculate the age-standardized incidence rate (ASR) was used from the world standard population. Data analysis was performed using SPSS software version 20 and at an error level of 5%.

**Results:**

The mean and standard deviation of age the injured was 32.9±18.8. The annually Incidence Rate (IR) of accidents and injuries was 939.1 cases per 100,000 people, while the age-standardized incidence rate (ASR) were estimated to be 917 per 100,000 people. In total, the sex ratio (male to female) in all accidents is 2.46, that this proportion in the age group of 20-29 years was maximum. The most incidents occurred in both sexes were reported motor vehicle (car) accident (32.2%), motorcycle accident (31.7%), pedestrian accident (16.2%) and poisoning (6.2%), respectively. Between gender variable and type of incident was observed significant difference (P<0.001). Cases of suicide attempt in the female group were 6.5 times higher than the men group.

**Conclusions:**

The incidence rate of traffic crashes in the city of Khomein is several times the provincial and national levels, for this reason, this city is considered as a high-risk and at-risk regions for traffic accidents in the country. Therefore, should be In addition to the optimizing the roads and removing the Accident-prone spots, The Naja Rahvar Police with by imposing strict and accurate traffic rules and more control of the drivers behavior, are attempting to reduce the occurrence of accidents. The high occurrence of poisonings and suicide attempt several times in the women s group is warning danger for the authorities to take preventive policies and applying cultural mechanisms to reduce this ratio to be stepped up.

**Keywords:**

Epidemiologic, Accidents and Injuries, IR, ASR, Suicide, Khomein

